# Comparison of the effects of different osteotomy methods on bone healing; an experimental study

**DOI:** 10.1186/s12891-026-09653-9

**Published:** 2026-03-10

**Authors:** Raşit Emin Dalaslan, Mehmet Arıcan, Zekeriya Okan Karaduman, Yalçın Turhan, Sönmez Sağlam, Mücahid Osman Yücel, Sinem Kantarcıoğlu Coşkun

**Affiliations:** 1 Orthopedics and Traumatology Department, Duzce University Medical Faculty, Konuralp street, Center, Duzce, 81000 Turkey; 2 Orthopedics and Traumatology Department, Bilkent City Hospital, Health Sciences University, Ankara, Turkey; 3 School of Medicine, Department of Pathology, Düzce University, Düzce, 81100 Turkey

**Keywords:** Bone healing, gigli wire, microsaw, drill and osteotome, osteotomy techniques

## Abstract

**Background:**

Bone healing after osteotomy remains a major issue in orthopedics. This study aimed to evaluate the radiological, histopathological, and biomechanical aspects of bone healing using three different osteotomy techniques.

**Methods:**

Fifty four Wistar Albino male rats were used. The animals were randomly divided into 3 groups as gigli (n:18), saw (n:18) and drill+osteotome (n:18). Transverse osteotomy was applied to the left femur with the gigli wire, microsaw and drill+osteotome. All femurs were evaluated radiologically, biomechanically and histopathologically at the end of the 15th, 30th and 45th days.

**Results:**

No significant differences in mean radiological and histological scores were observed among the gigli, saw, and drill+osteotome groups at most time points, except for a difference in radiological scores on day 30. The drill+osteotome group demonstrated more advanced callus formation, whereas the gigli group more often showed minimal or no callus. Biomechanical scores were lower in the gigli group on day 30, while no difference was observed between the saw and drill+osteotome groups.

**Conclusions:**

In the present study, the drill combined with an osteotome demonstrated superior biomechanical and radiological outcomes during the early phase of bone healing. However, in the later stages, all three osteotomy techniques exhibited comparable healing characteristics, suggesting that the initial advantage of the drill-assisted method may diminish over time.

**Supplementary Information:**

The online version contains supplementary material available at 10.1186/s12891-026-09653-9.

## Introduction

Osteotomy is a commonly performed procedure in orthopaedic and trauma surgery to correct congenital or post-traumatic deformities, as well as fractures that fail to heal in an anatomical position [1–5]. These procedures are typically carried out using surgical drills, saws, osteotomes, or gigli wires. However, the use of these instruments can lead to an increase in local temperature at the osteotomy site [1, 6]. Although mechanically rotating instruments are often equipped with internal cooling systems, complete prevention of thermal injury to the surrounding bone tissue remains challenging [7].

Bone healing after osteotomy is a multifactorial process influenced by various biological, mechanical, and environmental factors. Numerous pharmacological, physical, and biological approaches have been developed to enhance different stages of this process [6]. Experimental studies have indicated that cortical bone perfusion may be impaired during osteotomy due to thermal or mechanical factors. Localised heat-induced necrosis or microscopic sequestrum formation at the osteotomy level may predispose the site to infection and hinder physiological bone healing [1, 7].

Corrective osteotomies are successfully employed in orthopaedic and trauma surgery, particularly for congenital or post-traumatic deformities and joint-preserving procedures involving the hip, knee, ankle, and upper extremities [8–11]. To achieve complete bone healing, surgeons must take into account patient-related factors such as age, body mass index, and smoking status, as well as surgical parameters including osteotomy type, fixation method, osteotomy location, and the surgical instrument selected for the procedure [12, 13].

To date, no comparative studies have been reported that evaluate the use of gigli wire, saw, and drill combined with an osteotome, which are widely utilised in osteotomy procedures. Therefore, the present study aimed to evaluate and compare the radiological, histopathological, and biomechanical aspects of bone healing following osteotomies performed with a surgical saw, gigli wire, and drill combined with osteotome in a rat femur model. It was hypothesised that the osteotomy technique applied would influence the rate and quality of bone healing, with the drill combined with osteotome method providing superior early-stage outcomes compared to the other techniques.

## Materials and methods

This study was conducted using 54 male Wistar-Albino rats. Prior to the experiment, ethical approval was obtained from the Duzce University Faculty of Medicine Experimental Animals Local Ethics Committee (Approval No: 2021/02/06). All procedures were performed in the Experimental Animals Application and Research Center of the same institution in accordance with the principles of laboratory animal care. The rats were 2.5 months old on average (range: 2–3 months) and weighed approximately 250 g (range: 200–300 g). The sample size was determined a priori based on feasibility, ethical considerations (3Rs), and consistency with comparable femoral osteotomy studies, while allowing assessment at three predefined time points (15, 30, and 45 days) [14–16]. Accordingly, a total of 54 rats were included and randomly allocated into three groups: gigli wire (*n* = 18), saw (*n* = 18), and drill+osteotome (*n* = 18). Prior to the surgical procedure, all rats were acclimatized for one week under laboratory conditions, with six rats housed per cage. During the experiment, rats were provided ad libitum access to tap water and standard rodent chow. Environmental conditions were maintained at a controlled temperature (23–25 °C) with a 12:12-hour light/dark cycle. Preoperatively, antibiotic prophylaxis was administered to all groups (ceftriaxone, 50 mg/kg). No mortality occurred during the study period, and no wound infection was observed in any animal during follow-up. The study design, animal handling, experimental procedures, and outcome assessments were reported in accordance with commonly accepted standards for animal research reporting, including key principles of the ARRIVE guidelines.

After performing transverse osteotomy on the left femur of all rats using a gigli wire in the gigli group, a microsaw in the saw group, and a drill combined with an osteotome in the drill+osteotome group, the animals were sacrificed on the 15th, 30th, and 45th postoperative days. On postoperative day 15, six rats from each group were randomly selected and sacrificed. On day 30, another six randomly selected rats from each group were sacrificed, and on day 45, the remaining six rats in each group were sacrificed. The left femurs were then disarticulated from the hip and knee joints. Euthanasia was performed via an intraperitoneal overdose of sodium pentobarbital (Narcoren, Rhone Merienx) at a dose of 150 mg/kg, and death was confirmed by intracardiac puncture [17]. The soft tissues surrounding the femur were carefully removed without damaging the callus tissue, and the osteotomy sites were subsequently evaluated radiologically, biomechanically, and histopathologically.

### Surgical technique

**Fig. 1:**
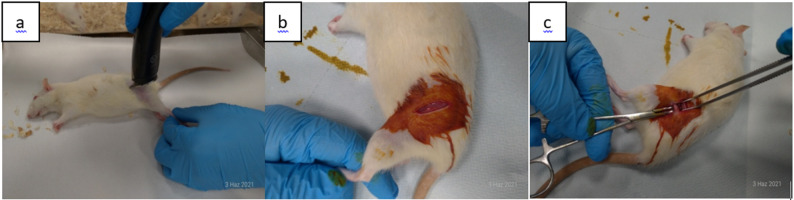
(**a**,** b**,** c**)**.** Surgical preparation process of rats femur before application. (**a**) Preparation of the thigh area by shaving, (**b**) Dissection of skin and subcutaneous muscles, (**c**) Exposure of the femoral shaft

The prepared rats were transferred to the surgical intervention room. Each rat was weighed using an electronic scale, and the anesthetic dose was calculated accordingly. A combination of ketamine (Eczacıbaşı, Istanbul, Turkey; 50 mg/kg) and xylazine (Bayer, Istanbul, Turkey; 10 mg/kg) was used for anesthesia, which was administered intraperitoneally from the right inguinal region. The left thighs of the rats were shaved and disinfected with povidone-iodine solution (Batticon^®^, ADEKA, Turkey). The knee and hip joints were palpated, and a 2 cm longitudinal skin incision was made on the lateral aspect of the mid-femoral shaft. After the fascia was incised with a scalpel, the muscles were bluntly separated, and the femoral shaft was exposed (Fig. 1a–c).

**Fig. 2:**
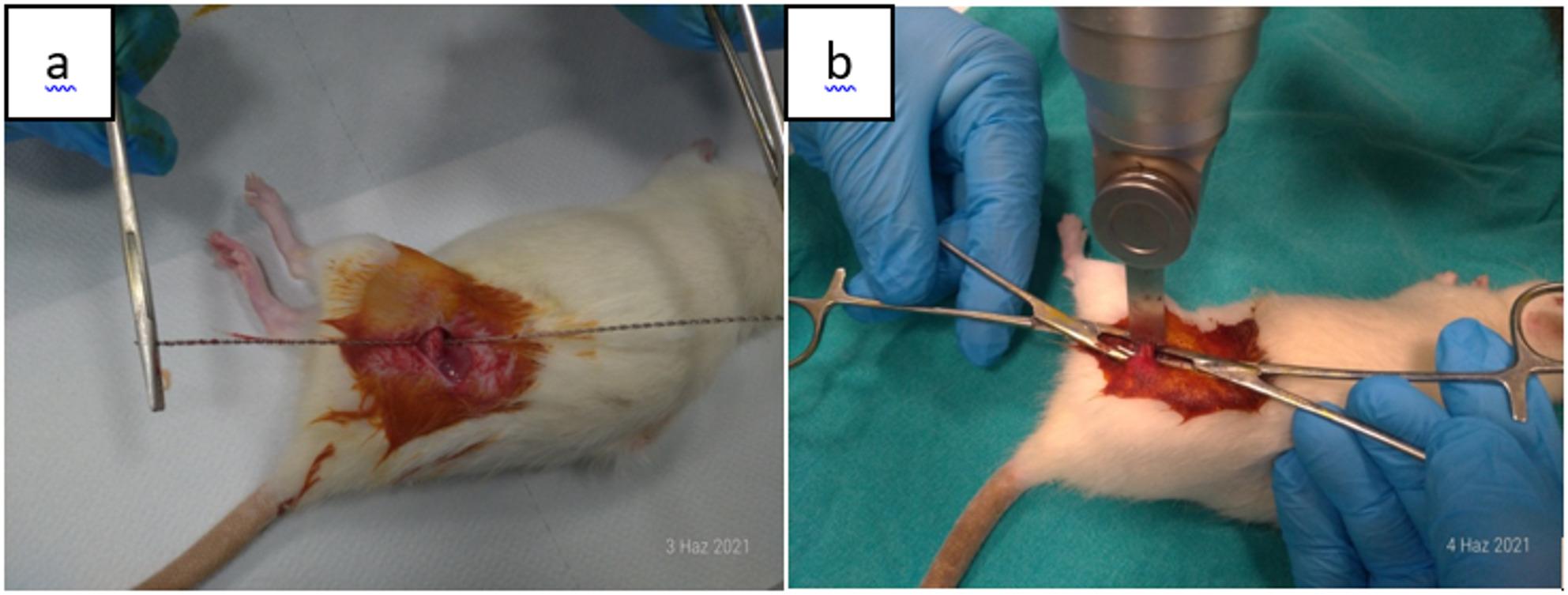
(**a**,** b**)**.** Application of osteotomy after exposure of the femur. (**a**) Performing osteotomy with a gigli wire, (**b**) Performing osteotomy with a saw blade

**Fig. 3:**
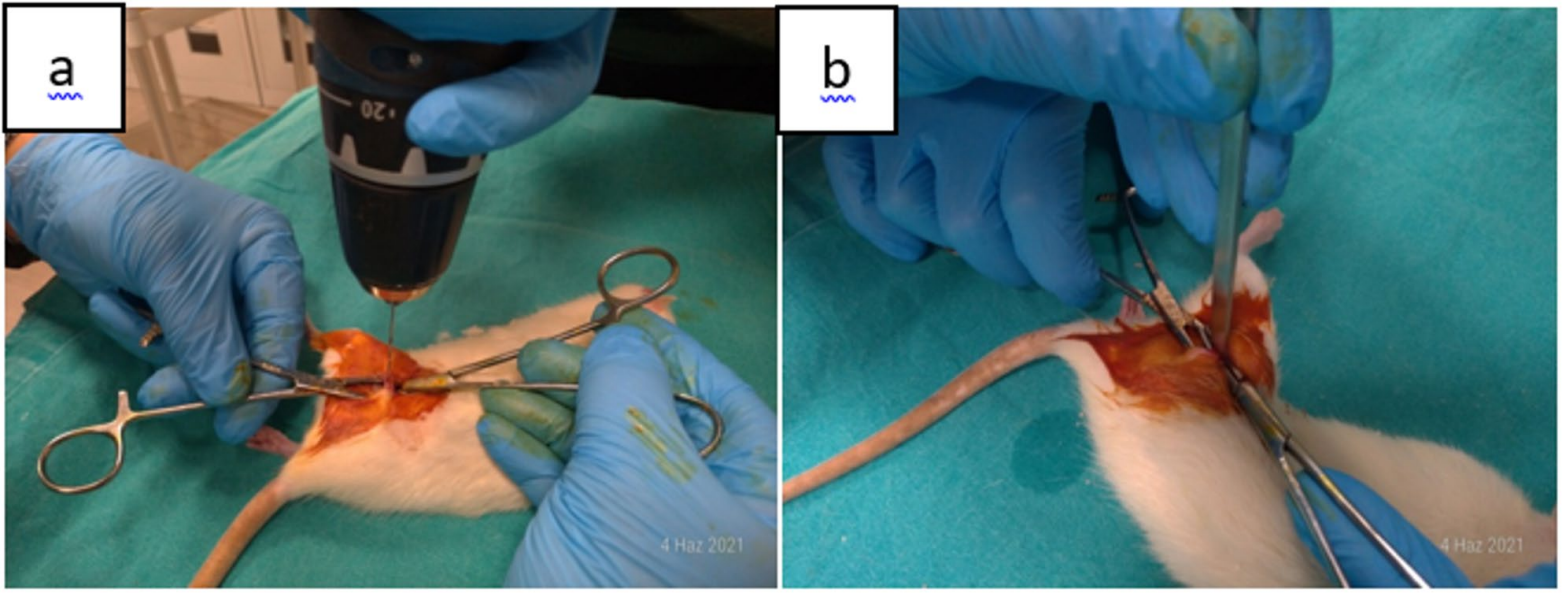
(**a**,** b**)**.** In the drill+osteotome group, osteotomy was performed with an osteotome after drilling from three different radial points

In the gigli group, after exposure of the left femur, a 0.66 mm gigli wire was positioned with the aid of a clamp, and a complete transverse osteotomy was performed with the wires placed at an approximate angle of 120° relative to each other, while carefully preserving the surrounding soft tissues. In the saw group, a standard transverse osteotomy was performed using a microsaw with a blade thickness of 0.8 mm **(**Fig. 2a, b**)**. In the drill+osteotome group, after exposure of the left femur, three pilot holes were created using a microdrill at the lateral, 45° anterolateral, and 45° posterolateral aspects to guide the osteotomy. The osteotomy line was then completed with an osteotome while maintaining soft tissue integrity (Fig. 3a, b).

All surgical procedures were performed by a single surgeon (RED). After the osteotomies in all groups, both fracture ends were stabilized with clamps. A 1.2 mm Kirschner wire (Tıpmed^®^, İzmir, Turkey) was inserted intramedullary into the distal fragment, exiting the knee joint. The motor end was then attached to the portion of the Kirschner wire protruding from the knee joint, and following reduction of the fracture, the wire was advanced intramedullary into the proximal femur, exiting the cortex. The portion of the wire remaining in the canal was trimmed at the level of the femoral condyles to prevent protrusion. The wire tip was left subcutaneously, the surgical site was disinfected with povidone-iodine, and the rat was removed from the operating table.

### Radiological evaluation

**Fig. 4:**
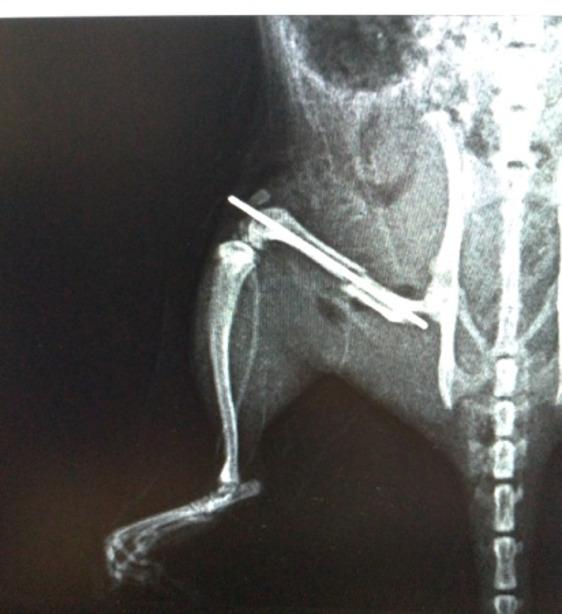
Postoperative control radiographs of the fixation material and osteotomy line were taken in all three groups

**Fig. 5:**
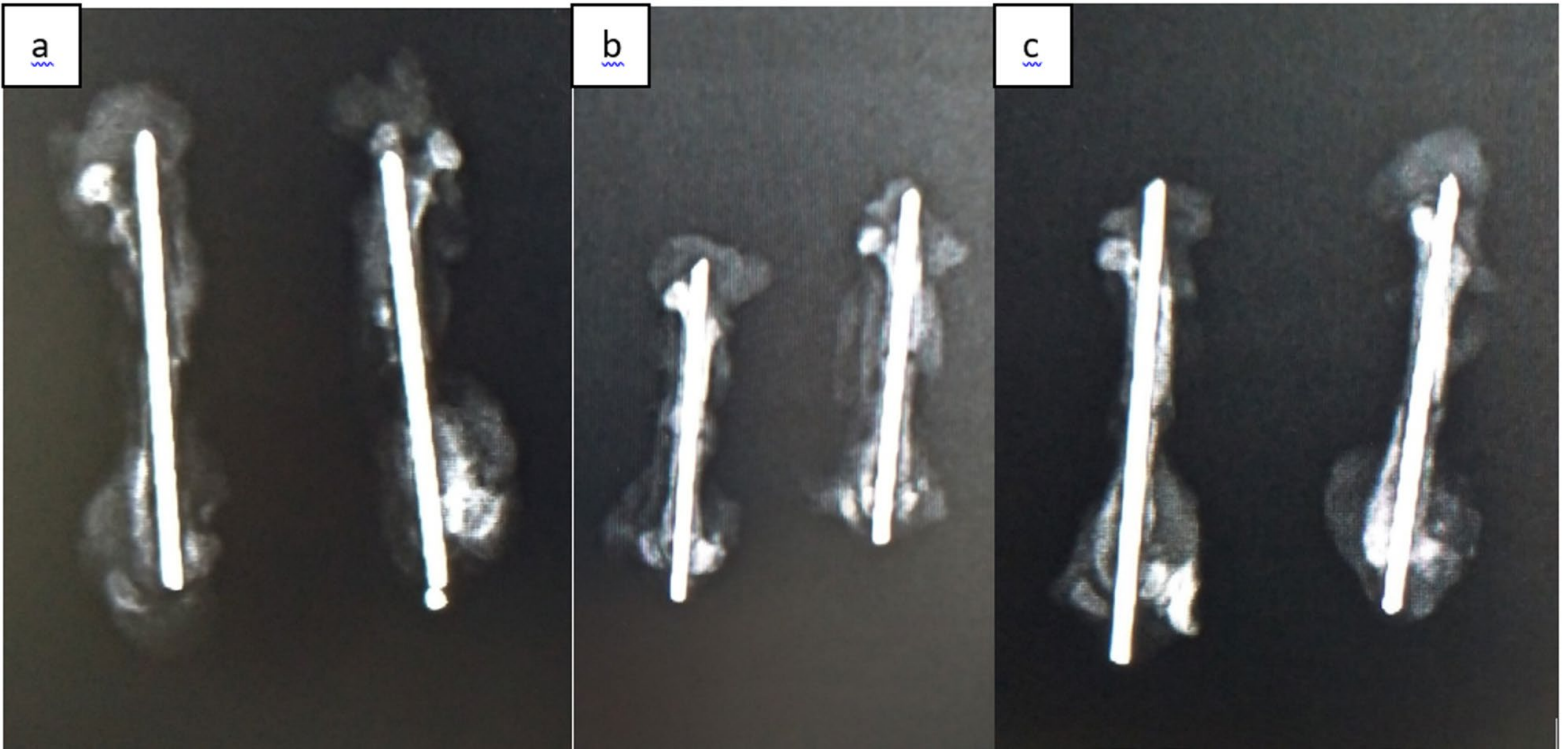
(**a**,** b**,** c**)**.** Control radiographs of the femurs of rats disarticulated after the sacrifice on the 15th day, (**a**) gigli wire group, (**b**) saw group, (**c**) drill+osteotome group

**Fig. 6:**
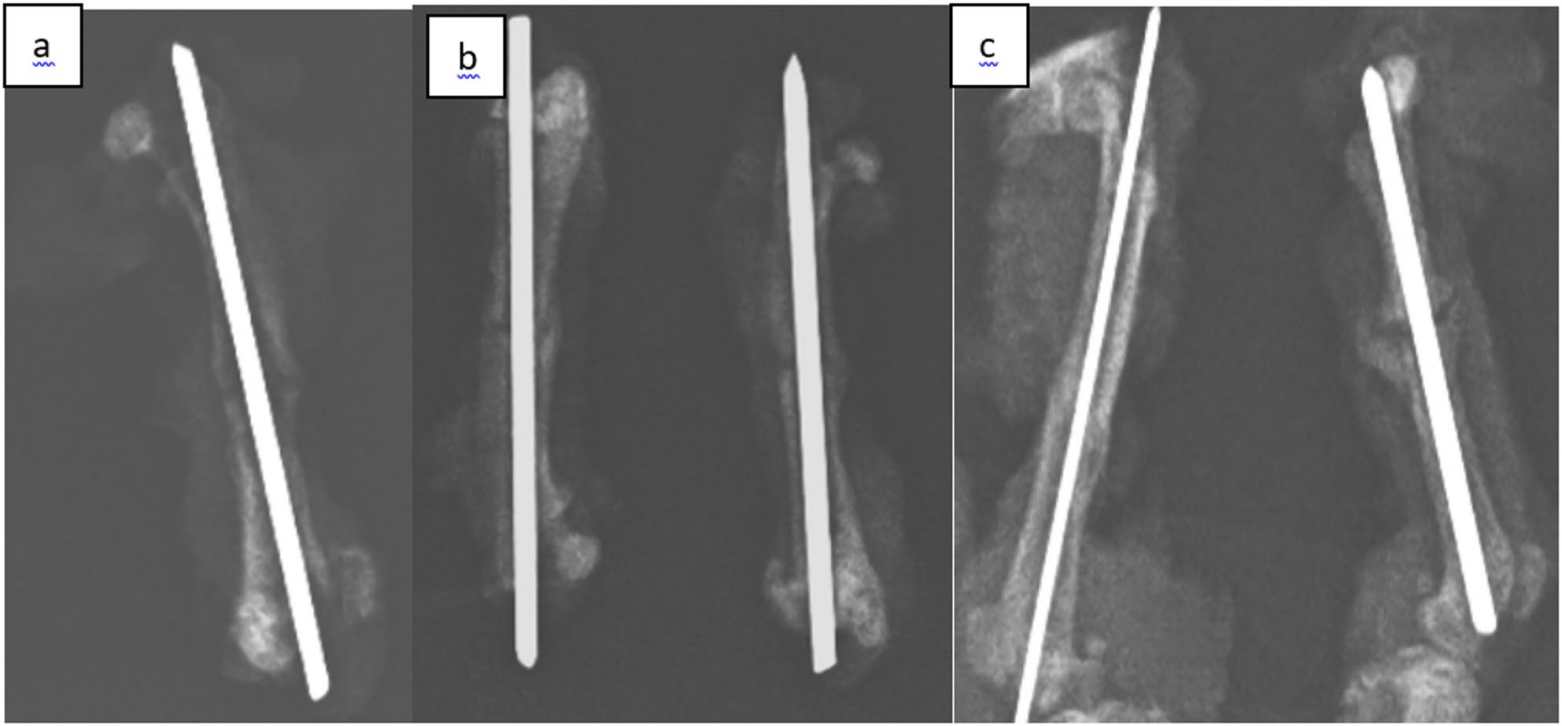
(**a**,** b**,** c**)**.** Control radiographs of the femurs of rats disarticulated after the sacrifice on the 30th day, (**a**) gigli wire group, (**b**) saw group, (**c**) drill+osteotome group

**Fig. 7:**
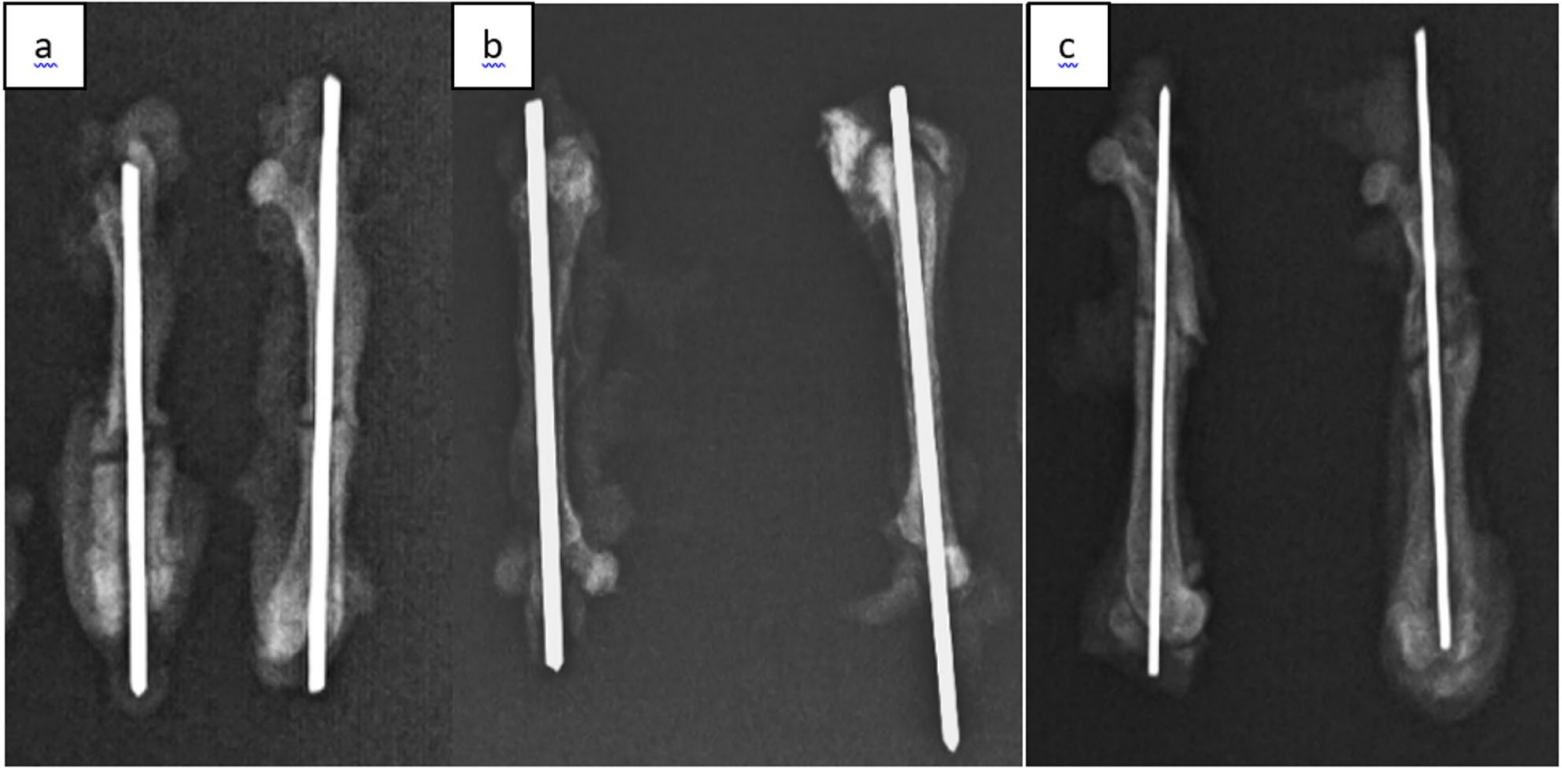
(**a**,** b**,** c**)**.** Control radiographs of the femurs of rats disarticulated after the sacrifice on the 45th day, (**a**) gigli wire group, (**b**) saw group, (**c**) drill+osteotome group

The osteotomy line and the fixation achieved after surgical intervention were confirmed radiologically using direct radiographs **(**Fig. 4**)**. Radiographs of the femurs were obtained from rats sacrificed on postoperative days 15, 30, and 45 in all three groups and were recorded for subsequent evaluation **(**Figs. 5a–c and 6a–c, and 7a–c**)**. Radiological scoring was performed using the grading system described by Lane et al. [16, 18] and was independently assessed by two orthopedic specialists (MA, YT). According to this scoring system, radiological fracture healing was graded on a scale from 0 to 4: 0 no callus formation, 1 minimal callus formation, 2 presence of callus but incomplete healing, 3 evident callus formation with expected stability, and 4 complete healing with bone remodeling.

### Biomechanical analysis

Biomechanical analysis was performed on 36 femurs from rats sacrificed on postoperative days 30 and 45 in the gigli, saw, and drill+osteotome groups. Although biomechanical testing was initially planned for day 15 samples, it could not be performed due to the presence of only soft callus. Analyses were conducted immediately after sacrifice without delay. The intramedullary fixation material was removed prior to testing.

**Fig. 8:**
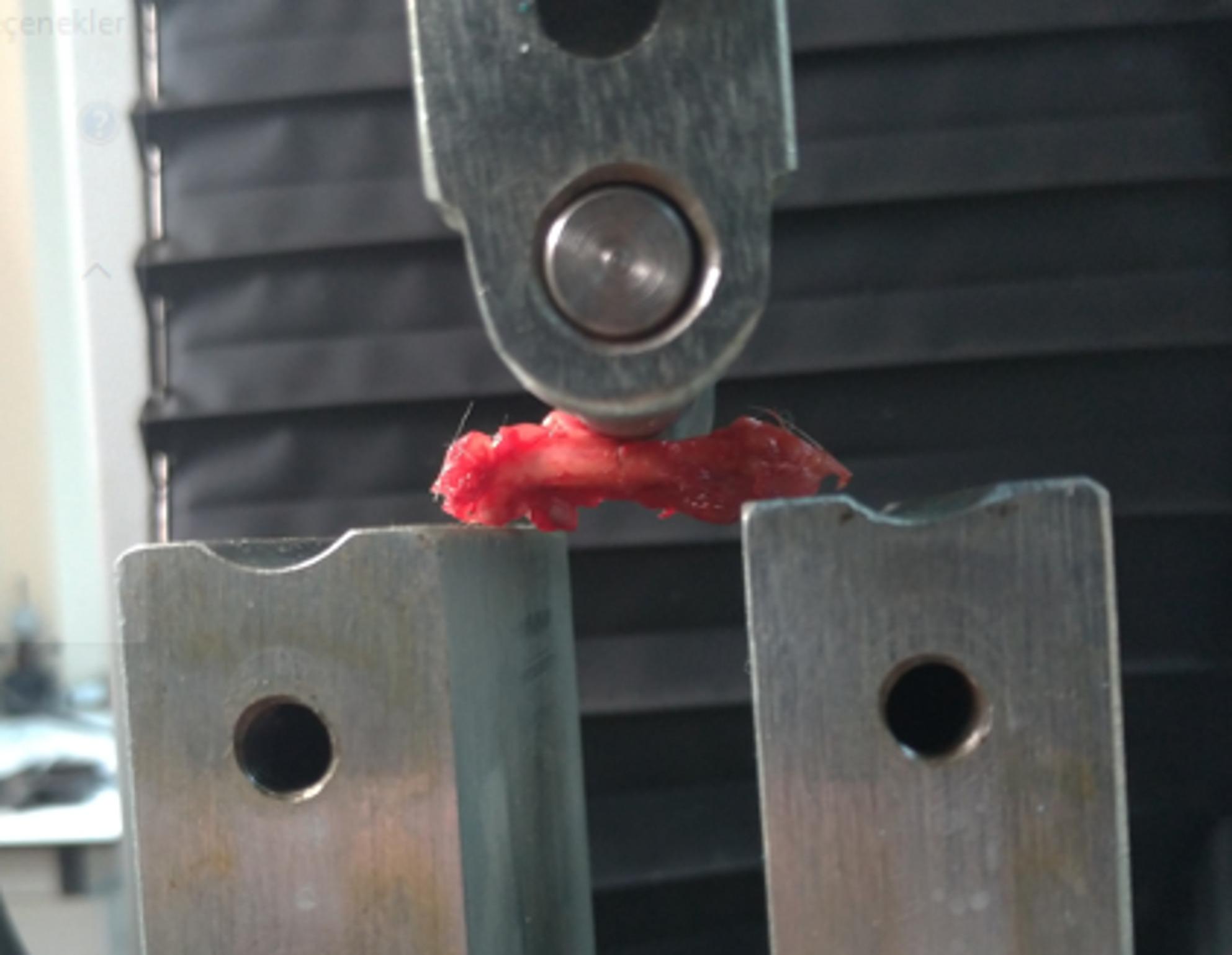
Performing three-point bending test for biomechanical analysis with BESMAK BMT-E Series material testing device

Biomechanical testing was carried out using the BESMAK BMT-E Series material testing device (Besmak, Ankara, Turkey) at the Duzce University Scientific and Technological Research Application and Research Center. A three-point bending test was employed for evaluation **(**Fig. 8**)**. The femurs were positioned horizontally on the testing apparatus with a support span of 18 mm, adjusted according to femur length. Bending force was applied at a rate of 3 mm/min, aligning the fracture line with the midline of the device. The force was increased until failure occurred, and the maximum force observed was recorded in newtons (N) for each sample.

### Histopathological evaluation

**Fig. 9:**
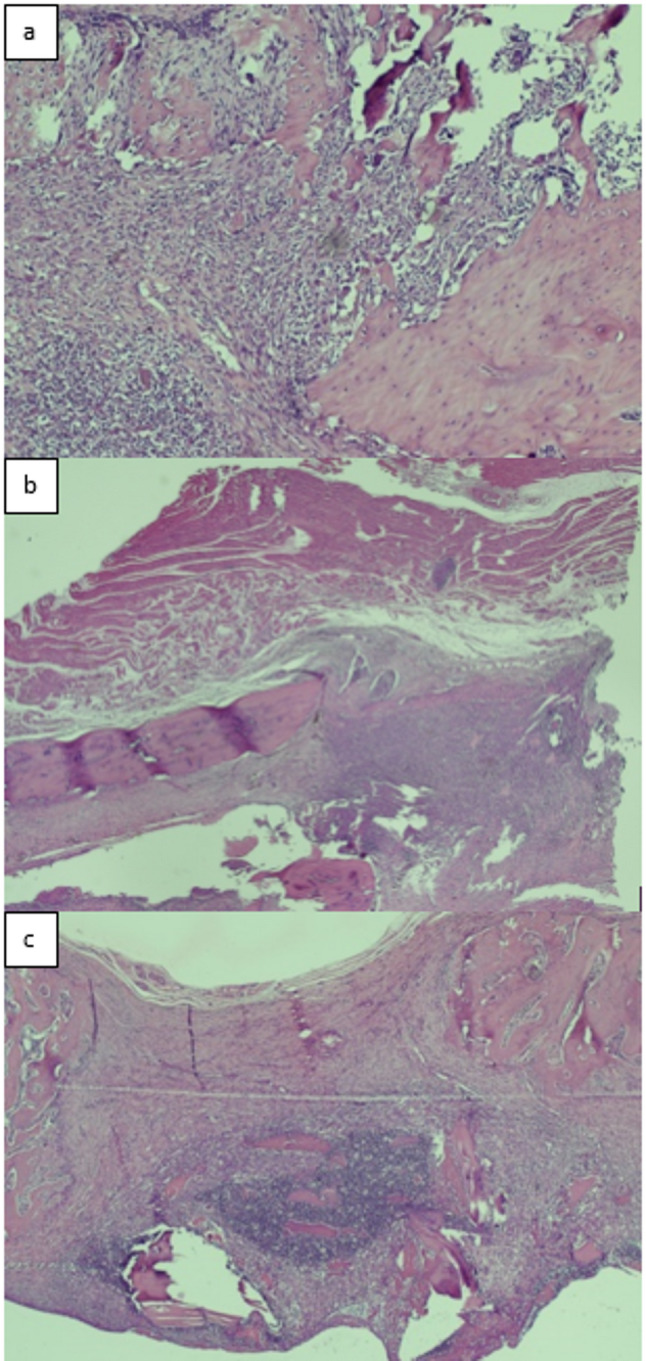
(**a**,** b**,**c**)**.** Histopathological evaluation of the gigli osteotomy group; (**a**) An example of fibrous tissue is seen from the histopathological appearance of the callus on the 15th day (score 1) (HE, x100), (**b**) An example of the histopathological appearance of the callus on the 30th day, showing fibrous tissue predominant and a small amount of cartilage (score 2) (HE, x2), (**c**) An example of the histopathological appearance of the callus on the 45th day shows immature bone and a small amount of mature bone (score 9) (HE, x4)

**Fig. 10:**
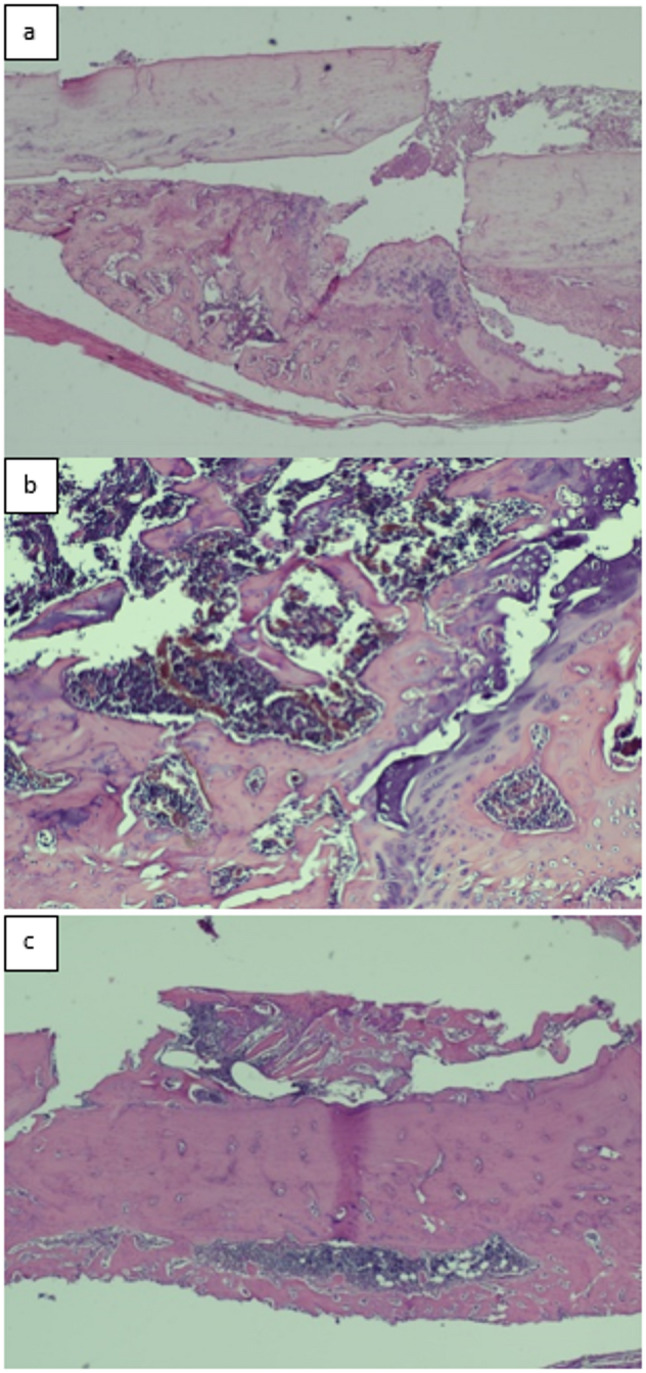
(**a**,** b**,**c**)**.** Histopathological evaluation of the saw blade osteotomy group; (**a**) An example of the histopathological appearance of the callus on the 15th day, showing predominantly immature bone and a small amount of cartilage (score 7)(HE, x4), (**b**) An example of the histopathological appearance of the callus on the 30th day, showing equal amounts of cartilage and immature bone (score 6)(HE, x10), (**c**) An example of mature bone is seen from the histopathological appearance of the callus on the 45th day (score 10)(HE, x4)

**Fig. 11:**
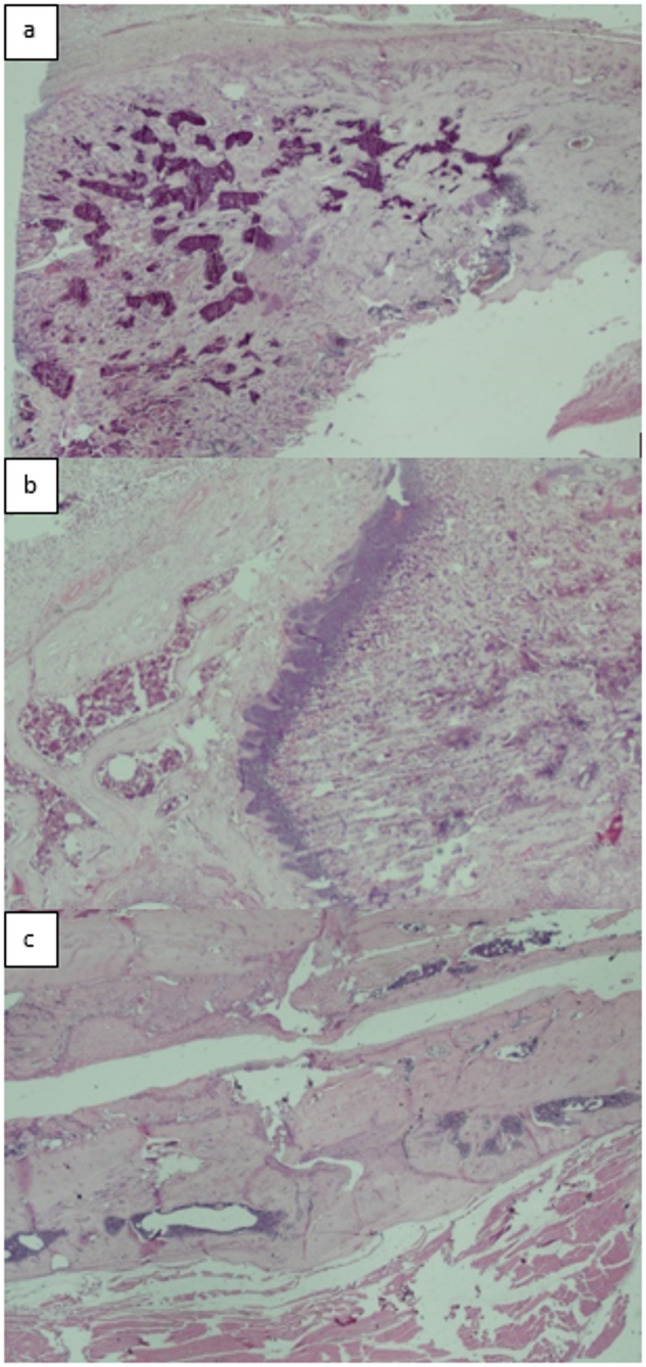
(**a**,** b**,**c**)**.** Histopathological evaluation of the group that underwent osteotomy with drill+osteotome (**a**) An example of the histopathological appearance of the callus on the 15th day, showing predominantly cartilage and a small amount of immature bone (score 5)(HE, x2), (**b**) A sample of cartilage is seen from the histopathological appearance of the callus on the 30th day (score 4)(HE, x4), (**c**) An example of the histopathological appearance of the callus on the 45th day, showing equal amounts of cartilage and fibrous tissue (score 3) (HE, x2)

All fractured femurs were carefully stripped of soft tissue while preserving the periosteum. Specimens were fixed in 4% paraformaldehyde at 4 °C for 48 h, decalcified in 7% formic acid, dehydrated, embedded in paraffin, and sectioned at 7 μm. Sections were stained with hematoxylin and eosin (H&E) and evaluated histopathologically using the scoring system described by Huo et al. [19], which semiquantitatively assesses fracture healing based on the predominant tissue type within the osteotomy gap. Lower scores (1–3) correspond to early stages of healing dominated by fibrous tissue and cartilage, intermediate scores (4–7) reflect the transition phase with increasing immature (woven) bone formation, and higher scores (8–10) indicate advanced healing with predominantly mature lamellar bone. This grading allows quantitative comparison of bone maturation and the progression of fracture repair among experimental groups. **(**Figs. 9a–c and 10a–c, and 11a–c**)**.

### Statistical analysis

Statistical analyses were performed using the NCSS (Number Cruncher Statistical System) 2007 software package (Utah, USA). Descriptive statistics, including mean, standard deviation, median, and interquartile range, were calculated. The normality of the data was assessed using the Shapiro-Wilk test. For variables that did not follow a normal distribution, intergroup comparisons were performed using the Kruskal-Wallis test, with Dunn’s multiple comparison test for post hoc subgroup analyses. Comparisons between paired groups were conducted using the Mann-Whitney U test, and qualitative data were analyzed with the chi-square test. Statistical significance was set at *p* < 0.05.

## Results

**Fig. 12:**
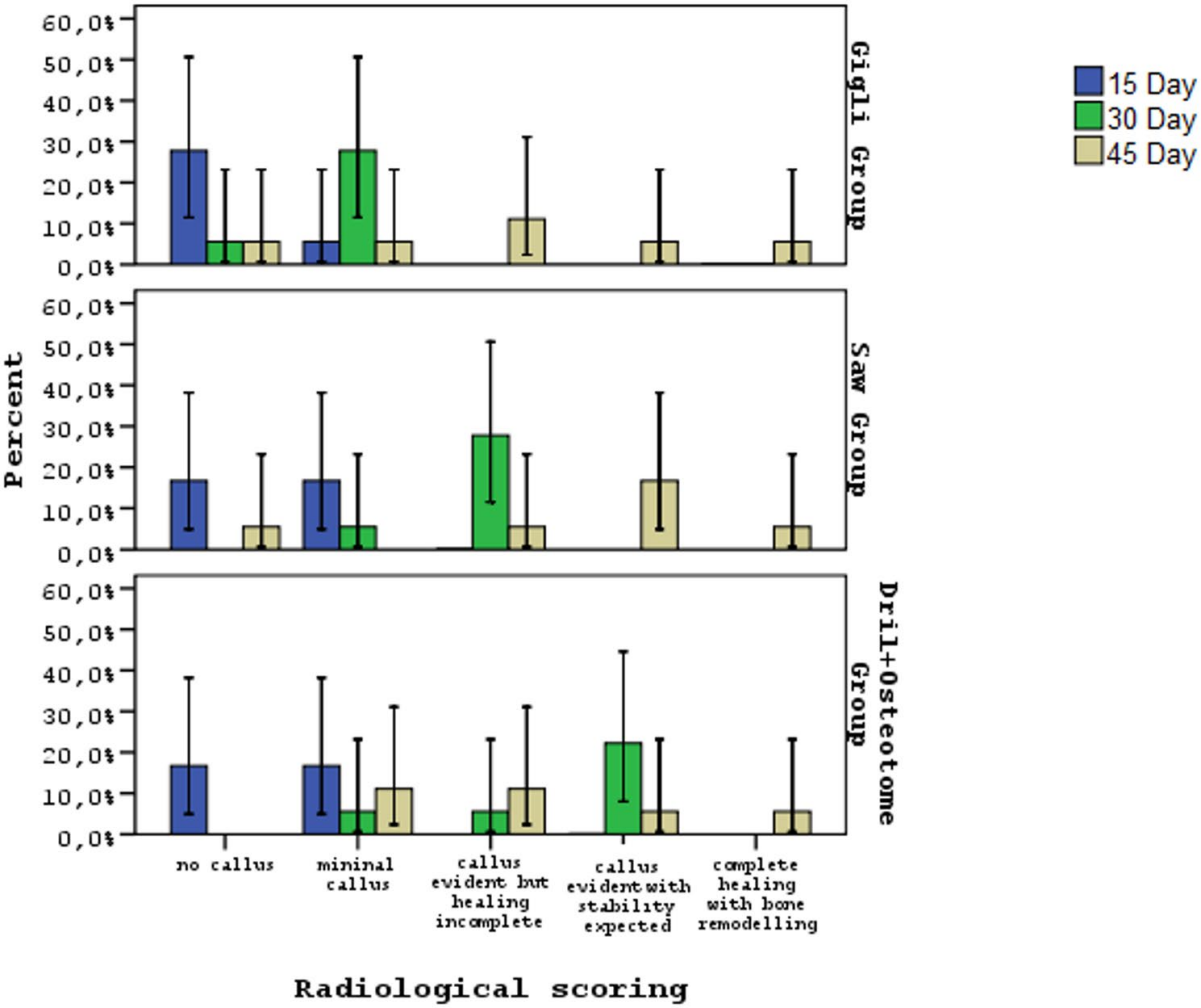
Evaluation of radiological scores of all groups

No statistically significant differences were observed in the radiological scoring distributions among the gigli, saw, and drill+osteotome groups on postoperative days 15 and 45 (*p* = 0.576, *p* = 0.889). However, a significant difference was found on day 30 (*p* = 0.001). In the drill+osteotome group, the category “callus evident with stability expected” was more frequent than in the gigli and saw groups, whereas in the gigli group, “no callus” and “minimal callus” were more prevalent compared to the drill+osteotome and saw groups. Across all groups, radiological scores changed significantly over time (days 15, 30, and 45; *p* = 0.018, *p* = 0.001, *p* = 0.037) **(**Table 1; Fig. 12**)**.

**Table 1:** Comparison of radiological scores of gigli, saw and drill+osteotome groups

Radiological Score		Gigli Group		Saw Group		Drill+Osteotome Group		*p*+
**Day 15**	**No Callus**	5	83,33%	3	50,00%	3	50,00%	0,576
	**Minimal Callus**	1	16,67%	3	50,00%	3	50,00%	
**Day 30**	**No Callus**	1	16,67%	0	0,00%	0	0,00%	**0,001**
	**Minimal Callus**	5	83,33%	1	16,67%	1	16,67%	
	**Callus evident but healing incomplete**	0	0,00%	5	83,33%	1	16,67%	
	**Callus evident with stability expected**	0	0,00%	0	0,00%	4	66,67%	
**Day 45**	**No Callus**	1	16,67%	1	16,67%	0	0,00%	0,889
	**Minimal Callus**	1	16,67%	0	0,00%	2	33,33%	
	**Callus evident but healing incomplete**	2	33,33%	1	16,67%	2	33,33%	
	**Callus evident with stability expected**	1	16,67%	3	50,00%	1	16,67%	
	**Complete healing with bone remodeling**	1	16,67%	1	16,67%	1	16,67%	
	**p+**	**0**,**018**		**0**,**001**		**0**,**037**		

Fisher’s exact test, Bold values indicate statistically significant differences (*p* < 0.05)

**Fig. 13:**
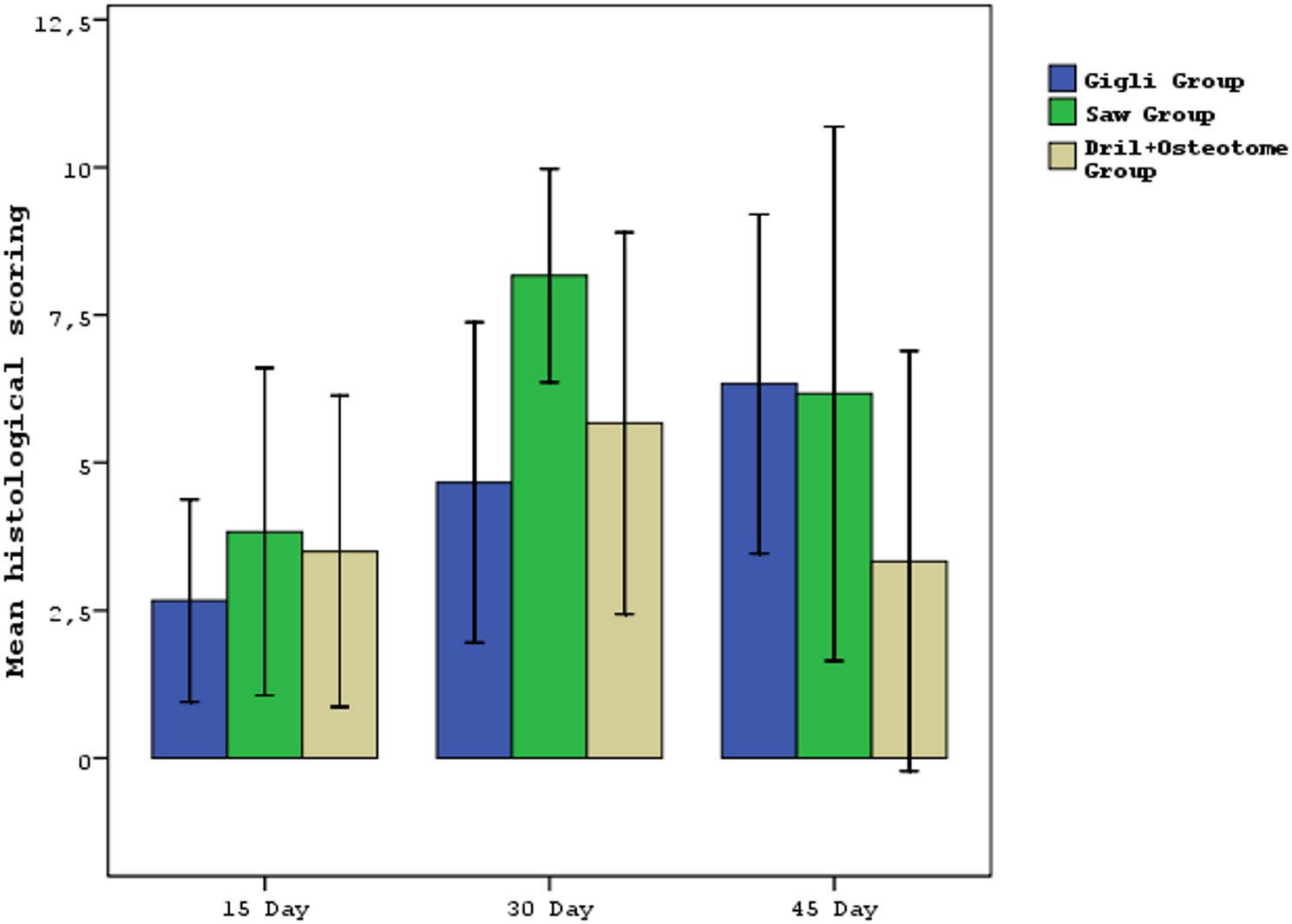
Evaluation of histopathological scores of all groups

Histological scoring did not differ significantly among the gigli, saw, and drill+osteotome groups on days 15, 30, or 45 (*p* = 0.719, *p* = 0.097, *p* = 0.352) **(**Table 2; Fig. 13**)**.

**Table 2:** Comparison of histopathological scores of gigli, saw and drill+osteotome groups

Histopathological Score		Gigli Group	Saw Group	Drill+Osteotome Group	p*
**Day 15**	**Mean ± SD**	2,67 ± 1,63	3,83 ± 2,64	3,50 ± 2,51	0,719
	**Median (IQR)**	2,50 (1–4,25)	3 (1,75 − 7)	3,50 (1–5,5)	
**Day 30**	**Mean ± SD**	4,67 ± 2,58	8,17 ± 1,72	5,67 ± 3,08	0,097
	**Median (IQR)**	5 (2–7)	8 (6,75 − 10)	4,50 (3–9,25)	
**Day 45**	**Mean ± SD**	6,33 ± 2,73	6,17 ± 4,31	3,33 ± 3,39	0,352
	**Median (IQR)**	7 (5,5–7,5)	7 (1,75 − 10)	2,50 (1–4,75)	
	p*	0,074	0,120	0,236	

**Kruskal Wallis test**,** SD; standard deviation**,** IQR; interquartile range**

Biomechanical evaluation revealed a significant difference among groups on day 30 (*p* = 0.013). The gigli group exhibited significantly lower biomechanical scores than both the saw and drill+osteotome groups (*p* = 0.026, *p* = 0.006), while no significant difference was observed between the saw and drill+osteotome groups (*p* = 0.810). On day 45, no significant differences were detected among the groups (*p* = 0.773) **(**Table 3**)**.

**Table 3:** Comparison of biomechanical scores of gigli, saw and drill+osteotome groups

Biomechanical Score		Gigli Group	Saw Group	Drill+Osteotome Group	p*
**Day 30**	**Mean ± SD**	7,67 ± 4,55	30,5 ± 15,44	32,83 ± 22,7	**0**,**013**
	**Median (IQR)**	5 (5–11,5)	31 (20,75 − 42,5)	25 (17–48,75)	
**Day 45**	**Mean ± SD**	20 ± 16,44	27,33 ± 20,02	27,5 ± 18,37	0,773
	**Median (IQR)**	15 (5–37,5)	30 (5–45,5)	32,5 (5–45)	
	**p‡**	0,145	0,871	0,936	
Dunn's Multiple Comparison Test (Day 30)	p				
Gigli Group / Saw Group	**0,026**				
Gigli Group / Drill+Osteotome Group	**0,006**				
Saw Group / Drill+Osteotome Group	0,810				

*Kruskal Wallis test, ‡Mann Whitney U test, *SD* standard deviation, *IQR* interquartile range, Bold values indicate statistically significant differences (*p* < 0.05)

## Discussion

In this study, the effects of three different osteotomy techniques, namely gigli wire, microsaw, and drill combined with osteotome, on bone healing were evaluated in a rat femur model. Radiological assessment revealed no significant differences among the groups on postoperative days 15 and 45, while a significant improvement in callus formation was observed in the drill+osteotome group on day 30. Histopathological analysis showed comparable healing patterns across all groups throughout the study period. Biomechanically, the drill+osteotome and saw groups exhibited superior resistance on day 30 compared to the gigli group, although no differences were noted by day 45.

Bone healing after osteotomy is affected by many factors related to surgery and non-surgical factors such as the method of osteotomy, fixation material and the area where the osteotomy is performed [20–26]. In our study, to minimize the influence of confounding factors, all procedures were performed on the same bone and at the same level in each rat, by the same surgical team, using an identical fixation method.

When the literature is reviewed, numerous clinical and experimental studies have investigated bone healing following osteotomy [20, 27–29]. In the study by Blaskovic et al. four different osteotomy techniques, including standard steel bur, piezosurgery (an ultrasonic vibration-based cutting device), and contact and non-contact erbium: yttrium-aluminum-garnet (Er: YAG) laser ablation, which are commonly used in maxillofacial surgery, were applied to rats. The resulting bone defects were examined by electron microscopy at the 1st, 2nd, and 3rd postoperative weeks. The initial examination performed immediately after surgery revealed that osteotomies created with a standard steel bur produced significantly more bone fragments and debris, whereas the other three methods yielded smoother and sharper osteotomy margins. Early bone formation within the defects was significantly faster in the piezosurgery group. Although all bone defects were completely filled by the 3rd postoperative week in all groups, initial bone healing was delayed in those performed with laser techniques. In particular, contact and non-contact Er: YAG laser modes resulted in delayed early healing, while piezosurgery accelerated the process; however, by the 3rd week, similar bone healing outcomes were observed across all groups [19].

In another study, Dumont et al. evaluated the long-term biomechanical and histological outcomes of fractures created by a saw compared to randomly induced fractures in sheep tibiae. A standardized 30° oblique osteotomy was performed on a single cortex using a saw, followed by manual completion of the fracture and plate fixation. Radiographs were obtained immediately postoperatively and at the end of the study. Bone healing was further analyzed by polychrome fluorescence labeling at 2, 4, 6, 10, 14, 18, 22, and 26 weeks in ten sheep. The three-point bending test revealed higher mean strength values in the operated bones compared to intact bones, although the difference was not statistically significant. Fluorescence analysis showed that saw osteotomies exhibited reduced vascularization within the osteotomy gap, as well as less fragment resorption and remodeling. In conclusion, Dumont et al. reported that bone healing after saw osteotomy was comparable to normal fracture healing, albeit with lower vascular density, fragment resorption, and callus remodeling [1].

Unlike that study, our experiment was limited to a 6-week observation period. Moreover, although the comparison groups differed, the biomechanical parameters of the saw osteotomy group in our study were superior to those of the gigli group and comparable to those of the drill+osteotome group.

The bone healing process after osteotomy may vary depending on the type of surgical instrumentation used. One of the main influencing factors is the thermal effect generated by different osteotomy techniques on bone tissue during the procedure [7, 13, 24, 26, 30, 31]. In an in vivo study by Gabric et al. temperature changes in bone tissue were measured using an infrared thermal camera during osteotomies performed in contact and non-contact modes with Er: YAG laser ablation, piezoelectric surgery, and a surgical drill. For each method, the baseline temperature before osteotomy and the maximum temperature during the procedure were recorded, and the average temperature change was calculated. The mean baseline temperatures (Tbase) were recorded as 27.9 ± 0.3 °C for contact Er: YAG laser, 29.9 ± 0.3 °C for non-contact Er: YAG laser, 29.4 ± 0.3 °C for piezosurgery, and 28.3 ± 0.3 °C for the surgical drill. The mean maximum temperatures were 29.9 ± 0.5 °C (ΔT = 1.9 ± 0.3 °C) for contact Er: YAG laser, 79.1 ± 4.6 °C (ΔT = 49.1 ± 4.4 °C) for non-contact Er: YAG laser, 29.1 ± 0.2 °C (ΔT = − 0.2 ± 0.3 °C) for piezosurgery, and 27.3 ± 0.4 °C (ΔT = − 0.9 ± 0.4 °C) for the surgical drill [20].

In another study, Gehrke et al. compared the effects of osteotomies performed using continuous or intermittent drilling modes and two different drill designs (cylindrical and conical) on thermal changes, bone healing, and polymorphonuclear cell counts. Osteotomies were performed using four techniques: continuous cylindrical drill, intermittent cylindrical drill, continuous conical drill, and intermittent conical drill. The mean temperature increases were 6.91 ± 1.4, 4.30 ± 1.3, 2.78 ± 0.6, and 2.77 ± 0.7 °C, respectively. The mean areas of new bone formation were 1.00 ± 0.3, 1.48 ± 0.3, 2.20 ± 0.4, and 2.43 ± 0.4 mm², while the mean polymorphonuclear cell counts were 62.4 ± 5.9, 50.7 ± 4.2, 44.4 ± 3.7, and 42.4 ± 3.7 cells, respectively. In both continuous and intermittent drilling modes, conical drills produced significantly lower temperature elevations, greater new bone formation, and fewer polymorphonuclear cells compared to cylindrical drills [13].

From a histopathological perspective, fracture healing progresses through four overlapping phases: inflammatory, soft callus formation, hard callus formation, and remodeling, corresponding to tissue transitions from fibrous tissue to cartilage, woven bone, and finally lamellar bone. The scoring system described by Huo et al. (1991) quantifies this progression, with higher scores indicating more advanced bone maturation [19]. Variations in osteotomy technique and thermal exposure can influence these stages, as excessive heat (> 47 °C) may induce osteonecrosis, reduce osteoblastic activity, and delay the replacement of cartilage by mineralized bone [32]. In our study, although direct temperature measurements were not performed, histological evaluation demonstrated similar progression patterns across all groups. Organized callus formation with active osteoblasts and new trabecular bone deposition was observed, with no significant necrosis or persistent fibrous tissue, indicating that none of the techniques generated heat or mechanical stress sufficient to disrupt normal healing. Mean histological scores in the gigli group increased steadily from 2.67 on day 15 to 6.33 on day 45, reflecting gradual maturation. The saw group peaked at 8.17 on day 30 and decreased to 4.17 on day 45, likely representing the transition from hard callus formation to remodeling, while the drill+osteotome group showed scores of 3.5, 5.67, and 3.33 on days 15, 30, and 45, suggesting early callus formation followed by remodeling. Given the experimental nature of the study and the limited number of animals evaluated at each time point, such variations in histological scoring are not unexpected and likely reflect biological variability rather than true impairment of bone healing. These findings indicate that, despite temporary variations in scoring potentially related to local mechanical and thermal effects, all osteotomy methods allowed normal histopathological progression of fracture healing.

Various shapes and techniques of osteotomy are used for deformity correction, bone lengthening, and bone transport, with the gigli wire and multiple drill methods among the most commonly employed [6, 29, 33–35]. Makhdoom et al. compared the modified healing index of these two methods in a retrospective clinical study including 50 adult patients who underwent tibial osteotomy for bone lengthening or transport using the Ilizarov circular fixator. Multiple drill osteotomy was performed in 23 patients, while gigli wire osteotomy was performed in 27 patients. The study reported that percutaneous gigli wire osteotomy, when performed through two small incisions, minimized local soft tissue trauma and periosteal disruption, resulting in superior consolidation during distraction osteogenesis compared to the multiple drill technique [29].

Similarly, Eralp et al. compared the healing indices of osteotomies performed with gigli wire and multiple drill methods in 44 tibiae of 41 patients undergoing lengthening with the Ilizarov method. Regardless of etiology, the mean healing index was 1.98 months/cm (range: 1.4–2.4) in the multiple drill group and 1.37 months/cm (range: 1.1–1.8) in the gigli wire group, confirming the biological advantage of the gigli wire technique [33].

In contrast, in our experimental rat model, the osteotomy line closed earlier in the drill+osteotome group compared to the gigli wire group. Biomechanical evaluation further demonstrated that femurs treated with the saw or drill+osteotome methods were stronger than those treated with gigli wire. This may be partly explained by the fact that rat femurs are considerably smaller than human bones, so thermal effects during drilling are likely minimal, which could have contributed to the earlier radiological and biomechanical advantages observed with the drill+osteotome technique. Similarly, the small working area in the rat model may have made it more difficult to prevent surrounding soft tissue from entering the osteotomy site in the gigli group, which could explain the relatively lower scores observed in this group. These findings suggest that, under controlled experimental conditions, drill+osteotome may provide earlier radiological and biomechanical advantages, although clinical outcomes in humans may favor minimally invasive gigli wire osteotomy due to soft tissue preservation.

There are some limitations to our study. First, our study is an animal model study and a short follow-up period was performed for bone healing. Second, bone formation and resorption markers were not examined as biochemical parameters. Thirdly, biomechanical examination was not performed due to insufficient callus tissue for 15 days during bone healing. Fourth, as intraoperative temperature measurements were not performed, the potential thermal effects of the procedures could not be adequately assessed.

In conclusion, the osteotomy technique applied influences bone healing. In our study, radiological and biomechanical outcomes at the end of the first month were superior in the saw and drill+osteotome groups compared to the gigli group. However, by the end of the study, complete bone healing was achieved in all three osteotomy methods. These findings suggest that while all techniques are capable of achieving fracture healing, the drill with osteotome method may offer earlier radiological and biomechanical advantages. Although this study was conducted in an animal model, further studies with longer follow-up periods, additional groups, or clinical settings are warranted to validate these results.

## Supplementary Information

Supplementary Material 1.

## Data Availability

The datasets used and/or analysed during the current study are available from the corresponding author(Mehmet Arıcan) on reasonable request.
